# Effect of mechanical loading on the metabolic activity of cells in the temporomandibular joint: a systematic review

**DOI:** 10.1007/s00784-017-2189-9

**Published:** 2017-08-01

**Authors:** Beatriz F. Betti, Vincent Everts, Johannes C. F. Ket, Hessam Tabeian, Astrid D. Bakker, Geerling E. Langenbach, Frank Lobbezoo

**Affiliations:** 1 Department of Orthodontics, Academic Centre for Dentistry Amsterdam, University of Amsterdam and VU University, Amsterdam, The Netherlands; 2 Department of Oral Cell Biology, Academic Centre for Dentistry Amsterdam (ACTA), University of Amsterdam and VU University Amsterdam, Amsterdam, The Netherlands; 3 Department of Oral Kinesiology, Academic Centre for Dentistry Amsterdam (ACTA), University of Amsterdam and VU University Amsterdam, Amsterdam, The Netherlands; 4 Department of Oral Kinesiology, Academic Centre for Dentistry Amsterdam (ACTA), University of Amsterdam and VU University Amsterdam, Amsterdam, The Netherlands

**Keywords:** Mechanical loading, Fibrocartilage, Temporomandibular joint, Cartilage degradation

## Abstract

**Objectives:**

The purpose of this systematic review was to elucidate how different modalities and intensities of mechanical loading affect the metabolic activity of cells within the fibro-cartilage of the temporomandibular joint (TMJ).

**Materials and methods:**

A systematic review was conducted according to PRISMA guidelines using PubMed, Embase, and Web of Science databases. The articles were selected following a priori formulated inclusion criteria (viz., in vivo and in vitro studies, mechanical loading experiments on TMJ, and the response of the TMJ).

A total of 254 records were identified. After removal of duplicates, 234 records were screened by assessing eligibility criteria for inclusion. Forty-nine articles were selected for full-text assessment. Of those, 23 were excluded because they presented high risk of bias or were reviews. Twenty-six experimental studies were included in this systematic review: 15 in vivo studies and 11 in vitro ones.

**Conclusion:**

The studies showed that dynamic mechanical loading is an important stimulus for mandibular growth and for the homeostasis of TMJ cartilage. When this loading is applied at a low intensity, it prevents breakdown of inflamed cartilage. Yet, frequent overloading at excessive levels induces accelerated cell death and an increased cartilage degradation.

**Clinical Significance:**

Knowledge about the way temporomandibular joint (TMJ) fibrocartilage responds to different types and intensities of mechanical loading is important to improve existing treatment protocols of degenerative joint disease of the TMJ, and also to better understand the regenerative pathway of this particular type of cartilage.

**Electronic supplementary material:**

The online version of this article (doi:10.1007/s00784-017-2189-9) contains supplementary material, which is available to authorized users.

## Introduction

The temporomandibular joint (TMJ) is covered by fibrocartilage, and its turnover depends on a balance between synthesis and degradation of the extracellular matrix (ECM). Synthesis of the ECM involves the production of collagen fibers, proteoglycans, and aggrecans, and its degradation is caused by the action of enzymes such as aggrecanases and matrix metalloproteinases (MMPs). An important mechanism responsible for the regulation of ECM turnover in the TMJ is mechanical loading [1, 2].

Two categories of mechanical loading can be discerned in the TMJ. The first is static loading, which occurs during teeth clenching, jaw bracing, and activities like swallowing. The second is dynamic loading, which occurs during tooth grinding, jaw thrusting, talking, and chewing. Bone and cartilage are responsible for transmitting and absorbing this mechanical loading [3, 4].

As cartilage is avascular, it needs to receive nutrients from the synovial fluid. This occurs by diffusion due to the movement of the fluid in and out of the cartilage matrix. This movement is caused by the cyclic mechanical loading of the joints (pumping). Pumping may also influence the diffusion of some solutes, such as growth factors, hormones, enzymes and their inhibitors, and cytokines towards the cells. In addition, cyclic mechanical loading helps the drainage of acidic waste materials, such as lactate and CO_2_ [5]. Future in vitro or finite element studies could elucidate the mechanism of activation of chondrocytes (i.e., direct transduction of mechanical signals to the chondrocytes vs. activation of chondrocytes by facilitated diffusion) in response to TMJ cartilage loading.

Thus, stimuli induced by mechanical loading can be highly beneficial for the maintenance and integrity of articular cartilage, as well as the development of the mandibular condyle [6].

While moderate dynamic loading is known to maintain the integrity of articular tissue during turnover and growth (anabolic effect), overloading can induce cartilage degradation (catabolic effect) [7]. It is not clear yet how these different loading intensities affect the TMJ cartilage, because in contrast with most synovial joints, which are covered by hyaline cartilage, the TMJ is covered by fibrocartilage. The collagen fibers contained in this TMJ fibrocartilage may provide some additional resistance against mechanical loading.

Knowledge about the way TMJ fibrocartilage responds to different types and intensities of mechanical loading is important to improve existing treatment protocols of degenerative joint disease (DJD) of the TMJ [8], and also to better understand the regenerative pathway of this particular type of cartilage. Therefore, we conducted this systematic review to find out how the TMJ fibrocartilage is affected by different modes of mechanical loading.

## Materials and methods

A review protocol was developed based on the Preferred Reporting Items for Systematic Reviews and Meta-Analysis (PRISMA) statement (www.prisma-statement.org). Embase.com, PubMed, and ISI/Web of Science were searched (by BFB and JCFK) from inception up to September 20th 2016 (see Supplementary information/Search strategy).

The following terms were used (including synonyms and closely related words) as index terms or free-text words: “bite force” or “shear stress” or “mechanical loading” and “cartilage” and ”temporomandibular joint.” The full search strategies for all the databases can be found in the Supplementary Information. Duplicate articles were excluded. Articles written in English were accepted.

The articles were selected by two independent authors (BFB and VE), following a priori formulated inclusion criteria (viz., in vivo and in vitro studies, mechanical loading experiments on TMJ, and the response of the TMJ). After a subsequent analysis of confounding factors and quality of the research design, papers with sufficient quality were finally selected for this review.

## Results

### Literature identification

**Fig. 1:**
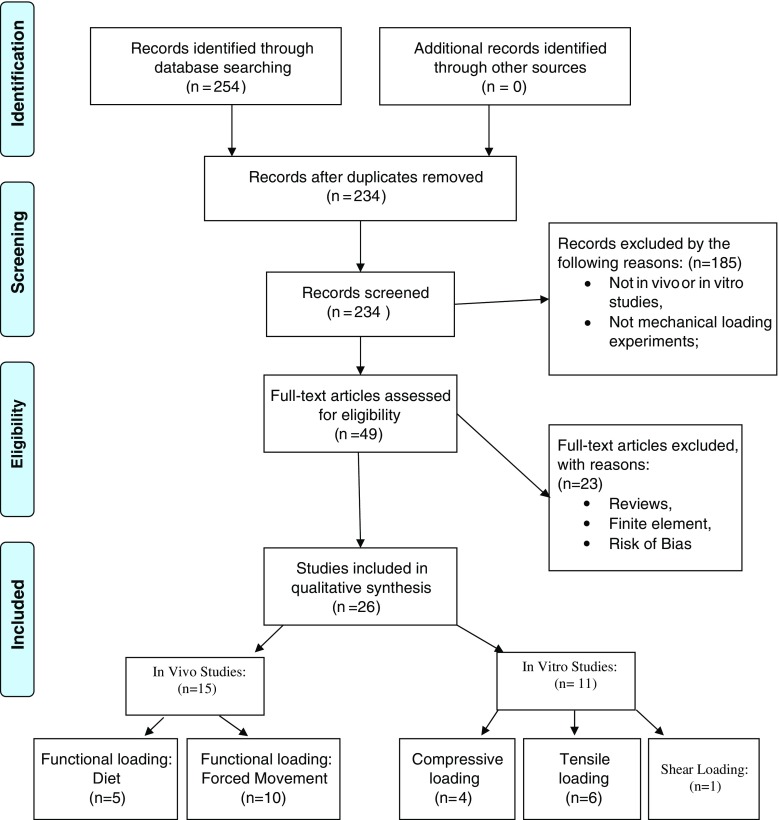
PRISMA flow chart: The flow describes the information through the different phases of a systematic review. It maps out the number of records identified, those included and excluded, and the reasons for exclusions

With the above-described literature search strategy, 254 records were identified. The complete inclusion process is shown in Fig. 1. No additional records were identified through other sources. After removal of duplicates, 234 records were screened by assessing eligibility criteria for inclusion.

Forty-nine articles were selected after the eligibility inclusion and exclusion criteria for a full-text assessment. Of those, 23 articles were excluded for the following reasons: reviews of experimental studies or abstracts (*n* = 12), a finite element study (*n* = 1), or presence of risk of bias (*n* = 10) (Tables 1 and 2).

**Table 1:** Risk of bias (exclusion criteria)

Study	Reason for exclusion
Pirttiniemi et al. year (1996)	Lack of proper controls^a^
Herring et al. year (2002)	Possible local differences in loading were not analyzed. The study can only be used to indicate the site of proliferation but does not show the effect of loading on proliferation.
Wattanachai et al. (2009)	Lack of proper controls^b^
Fujimura et al. (2005)	Lack of proper controls^b^
Pirttiniemi et al. (2004)	Lack of proper controls^a^
Tuominen et al. (1996)	Lack of proper controls^a^
Magara et al. (2012)	Lack of proper controls^b^
Wen et al. (2016)	Lack of proper controls^d^
Henderson et al. (2015)	Lack of proper controls^v^
Lin. H et al. (2009)	Lack of proper controls^2^

^a^The intervention should have been applied to both diet groups: soft and hard diet

^b^A sham-operated group should have been added as control

^c^Unilateral splint could affect the non-loaded joint; a control without splint should have been added

^d^Lack of a control group with an injection of salubrinal but without loading

**Table 2:** Characteristics of the included studies

Study	Study design	Sample	How loading was applied	Where the effects were looked for	Main findings	Conclusions
J.C. Nickel et al. 2004 [9]	In vitro	50 TMJ discs from mixed-breed pigs	Static compressive loading: EG1 (10 N, 10 s) EG2 (10 N, 60 s)	Disc mechanical properties: Maximum tractional force, maximum compressive stress, peak stress	EG1: max tractional force EG2: max compressive stress	The magnitudes of forces and compressive stresses produced on the surface of the disc depended on duration of pre-loading.
G.D. Nicodemus et al. 2007 [10]	In vitro	5 bovine heads, TMJ cell isolation	Dynamic compressive loading: CG (unloaded) EG (15% strain) EG1 (24 h) EG2 (48 h)	Cellular response: Collagen type I, collagen type II and aggrecan gene expression	Gene expression of Coll I, II, and aggrecan: CG = EG1 ˃ EG2	Dynamic compressive strains resulted in inhibition of gene expression, cell proliferation, and proteoglycan synthesis.
M.J. Ravosa et al. 2006 [11]	In vivo	20 rabbits CG (10) EG (10)	Functional loading: CG (soft diet) EG (hard diet)	Condyle structure: Collagen type II, apoptotic chondrocytes	EG: increase of Col II and number of apoptotic chondrocytes	Compensatory mechanism to cartilage degradation serves to maintain the overall functional integrity of each joint.
K.Fujimura et al. 2005 [12]	In vivo	30 rabbits CG (06) EG (24)	Functional loading: CG (unloaded) EG (100 g force applied by a coil spring) EG1 (1 week) EG2 (2 weeks) EG3 (4 weeks) EG4 (8 weeks)	Condyle structure: Collagen type II and histological synovitis score	Synovitis begun 1–2 weeks after loading started Collagen type II decreased first at the articular eminence and after at the condyle	Mild, continuous mechanical loading of the glenoid fossa induces synovitis of the articular capsule and induces organic changes of the articular cartilage but not the degradation of these tissues.
T. Soube et al. 2011 [13]	In vivo	48 mice CG (16) EG (32)	Functional loading: CG (unloaded) EG (1 h continuous forced month opening/day EG1 (25 N) EG2 (50 N)	Cellular response: Gene expression (collagen type I and II, PTHrp and sox9) Condylar structure: Increasing of trabecular space	EG1: no significant changes EG2: increase gene expression and increase of the trabecular spacing in the subchondral bone	Forced mouth opening causes increased expression of mandibular chondrocyte maturation markers and decrease in the subchondral bone volume.
N. Hichijo et al. 2014 [4]	In vivo	14 rats CG (7) EG (7)	Functional loading: CG (normal diet) EG (soft diet)	Cellular response and condyle structure: Cartilage thickness, IGF-1r expression	EG: reduction of the cartilage thickness, and reduction of IGF-1r immune positive cells	A decrease in masticatory demand during the growth period leads to insufficient mandibular development, decreasing the IGF-1r expression and cartilage thickness.
W. Chen et al. 2013 [2]	In vitro	Rats Isolated mandibular cartilage cells	Dynamic compressive loading: CG (unloaded) EG (2000, 4000, 6000 μ strain for 6,12 and 24 h) EG1–9	Cellular response: Collagen and proteoglycan synthesis plasminogen activator (PA) activity	EG 2000 and 4000: increase of Collagen and proteoglycans synthesis, and low PA activity EG 6000: decrease of proteoglycans and collagen synthesis and increase of PA activity	Mechanical overload upregulated PA activity, providing a proteolytic environment of extracellular matrix components and contributing to cartilage degradation in TMJ osteoarthritis.
D. Yu et al. 2007 [1]	In vivo	100 rats CG (50) EG (50)	Functional loading: CG 1–5 (soft diet during 6,12,24 and 48 h and 9 days) EG 1–5 (hard diet during 6,12,24 and 48 h and 9 days)	Cellular response: Immunohistochemical (IHC) analysis and western blot (WB) aggrecanase-1 and TIMP-3	EG (only on IHC, no difference was found on WB) aggrecanase-1 was higher at 12 and 24 h, after 48 h, there was no difference. TIMP-3 was lower at 6 h	Temporary increases in aggrecanases-1 and TIMP-3 occurred in the hard diet group, showing the complex cartilage response during altered dietary loading.
Y.-D. Liu et al. 2014 [14]	In vivo	40 rats CG (20) EG (20)	Functional loading: CG1 (small size diet) CG2 (large size diet) EG1 (small diet + anterior cross-bite prosthesis) EG2 (large diet + anterior cross-bite prosthesis)	Cellular response and condyle structure: -Thickness; - Collagen type II, aggrecan and ADAMTS-5-osteoclastic activity	CG1 and CG2: no difference on thickness and TRAP(osteoclast) EG1 and EG2: decrease the cartilage thickness, but 2 more than 1 . EG1 and EG2: Increase osteoclast activity but 2, more than 1. EG1 and EG2: Col II and Aggrecan gene expression decrease in both groups	Lower level of functional loading by providing small-size diet could reduce TMJ degradation induced by biomechanical stimulation from abnormal occlusion.
A. Poikela et al. 2000 [15]	In vivo	86 rabbits CG (43) EG (43)	Functional under loading: CG1 = no grinding 25 days CG2 = no grinding 35 days EG1 = unilateral grinding molars right side, twice a week. 25 days EG2 = unilateral grinding molars right side, twice a week. 35 days	Cellular response: Histological analyses (Safranin O staining) Contents and distribution of proteoglycans in the condyle cartilage	25 days rabbits: Proteoglycans amount Right condyle EG1˂CG1 Left condyle EG1 = CG1 35 days Rabbits: Proteoglycans amount Right and Left Condyles EG2˂ CG2	The mechanical properties of the articular cartilage after a period of unilateral mastication was impaired, and it is possible that this makes the joint cartilage more susceptible to pathological events.
T. Fujisawa et al. 2003 [16]	In vivo	9 rabbits CG1 (1) CG2 (1) Radiographic control (1) EG1 (3) EG2 (3)	Functional over loading: CG1 = no loading (1 day) CG2 = no loading (7 days) EG1 = steady mouth- opening 3 h/day (1 day) EG2 = steady mouth opening 3 h/day (7 days)	OA-like lesion at TMJ condyle: Macroscopic and histological	Macroscopic: CG1 and 2: no damage EG1 and 2: articular surface fibrillation (roughness) and some subchondral bone exposures EG2˃ EG1 Histopathological: CG1 and 2: normal histology EG1: thinning of the articular cartilage EG2: OA-likes lesions (complete loss of the articular cartilage)	Repetitive, forced-jaw-opening can induce OA-like lesions.
M. Orajävi et al. 2012 [11]	In vivo	36 rats CG1 (8) CG2 (8) EG1 (10) EG2 (10)	Functional under loading + hormonal: CG1 = non-ovarectomized + normal diet CG2 = non-ovarectomized + soft diet EG1 = ovarectomized + normal diet EG2 = ovarectomized + soft diet	Cell response and histomorphometric: Number of cells, cartilage thickness, Col II, and MMP-3 gene expression	Histomorphometric Cartilage thickness: EG1˃CG1 Number of cells: EG2˃CG2 CG1 = CG2 Col II: EG1 /2 ˃ CG1/2 EG1/2 ˃ CG1/2 MMP-3: EG1/2 ˃CG1/2	Condylar cartilage is sensitive to both estrogen level and mechanical loading, i.e., estrogen reduced MMP-3 expression and a soft diet enhanced the area covered by collagen type II and X.
M. Zhang et al. 2016 [17]	In vivo	160 rats CG1–5 (16 each group) EG1–5 (16 each group)	Functional over loading: CG (unloading) EG (anterior cross bite for 2,4,6,8,12 and 20 weeks)	Tissue response: Calcified cartilage thickness,	EG(2 Weeks): ↑ collagen fibers and hypertrophic chondrocytes EG (2,8 Weeks): ↓ Chondrogenic markers: Col-2, X and aggrecan EG (12,20 Weeks): mineral deposits in TMJ cartilage	Light forces provide benefits for TMJ remodeling while heavy force induce degenerative process on the TMJ.
Y. Ikeda et al. 2014 [18]	In vivo	40 rats CG (10) EG1 (10) EG2 (10) EG3 (10)	Forced mouth opening: EG1 = mouth open EG2 = liquid diet feeding EG3- = mouth open + liquid diet feeding	Tissue response: Cartilage thickness, MMP-13	EG3: decrease trabecular thickness and MMP-13 was higher than the other groups	TMJ hypofunction leads to OA-like changes when also exposed to mechanical over loading.
Zhang.C et al. 2015 [19]	In vivo	232 rats CG EG Light force (1–8) EG2 heavy force (1–8)	Functional over loading: Forced unilateral movements (light and heavy force for 3, 7, 14, and 28 days and rest for 3,7,14 and 28 days)	Tissue response: Cartilage thickness	On the loading side: EG heavy force: cartilage thickness on the anterior part of condyle decrease on the force period and increase during the recovery period, and on the medium and posterior parts the other way around EG light forces: showed the same but was not significant different	Asymmetric heavy force damages the cartilage and light forces provide remodeling responses.
S. Kartha et al. 2016 [20]	In vivo	Rats (number of sample not given) CG1 CG2 EG1 EG2	Functional over loading: Forced mouth opening (7 days loading +7 rest days) CG1 = no loading EG1 = 2 N force EG2 = 3,5 N force2	Tissue response: Densitometry and IHC Cellular response: MMP-13, HIF-1α and TNF-α	EG1 and EG2: showed OA like lesions EG2: ↑MMP-13, HIF-1α and TNF-α	The upregulation of the cellular markers could predict the maintenance of orofacial pain and TMJ degradation.
H.J. Yang, S.J. Hwang 2014 [21]	In vivo	Rabbits (15) CG (03) EG1 (06) EG2 (06)	Functional changing of loading: Unilateral osteotomy of the mandible and counter clock-wise rotation CG = no surgery EG1 = 1 mm rotation of proximal segment EG2 = 3 mm rotation of the proximal segment	Tissue response: Micro CT and histological evaluation	EG1 and EG2: Osteoporotic changes of TMJ condyle (↓bone volume and bone mineral density) ↓cartilage thickness	Changing loading direction can cause a different area of compression/tension/shear of the condyle, leading to degradation.
A. Utreja et al. 2016 [8]	In vivo	12 Mice CG (06) EG (06)	Functional over loading: forced mouth opening: CG (no loading) EG (1 h loading for 5 days)	Cellular response: Cell maturation by fluorescent reporters (DKK3, ColI, ColII, ColX)	EG: DKK increased at superficial zone. ColI and II increased at pre hypertrophic zone. ColX increased at hypertrophic zone.	TMJ cartilage responds to static loading by forming thicker cartilage through adaptive remodeling
S. Fazeli et al. 2016 [22]	In vitro	5 pig TMJ discs CG (left disc) EG (right disc)	Compressive loading after collagenase CG (loading, no treatment) EG (loading after collagenase)	Biomechanical and tissue response: Collagen and GAG content Collagen fiber alignment	EG: Compressive moduli decreases at 50–90% lower collagen and GAG content	Disruption of collagen fibers can lead to mechanical softening of TMJ discs decreasing their mechanical stability under compression
R.S. Carvalho et al. 1995 [23]	In vitro	48 rats CG (12) EG1 (12) EG2 (12) EG3 (12)	Compressive over loading: CG = No loading EG1 = extensive intermittent compressive loading EG2 = moderate intermittent compressive loading EG3 = continuous compressive loading Ages of 7 and 9 weeks (24 rats in each group	Tissue response: Amount of GAG	No differences in GAG amount between ages EG2 increased chondroitin sulfate	Compressive forces in the articular disk may stimulate the development of more cartilaginous-like properties with respect to GAG components
C.M. Juran et al. 2013 [24]	In vitro	Porcine fresh TMJ disc cartilage	Compressive + shear loading: CG (no loading/ control group) EG1 (loading at anterior part of the disc) EG2 (loading at intermediate part of the disc) EG3 (loading at posterior part of the disc) 27 testing procedures [frequency variation (0.5, 1 and 5 Hz), compressive strain (5, 10, 15%) and shear strain variation (1, 3 and 5%)	Disc structure: Cartilage fatigue and damage	EG3: maintain stiffness after compressed and sheared loading EG1 and EG2: decrease the stiffness after compressed and shear loading	The mechanical characteristics of the TMJ disc are highly dependent on the ECM microenvironment and its regional composition.
Y.-Y. Lin et al. 2009 [25]	In vitro	Porcine fresh TMJ condyle punch cartilage + bone CG(left condyle) EG (right condyle)	Compressive impact loading: CG = No Loading EG = 200 g mass was dropped from a height of 60 cm onto the top of the holding condylar heads	Cellular response: Il-1β, Col II (Cartilage) Il-1α and IL-1β (Subchondral bone)	IL-1β: EG˃CG (cartilage and bone) Col II: EG˃ CG (chondrocytes) IL-1β and IL-1α: EG˃ CG (subchondral bone)	Impact loading can increase directly IL-1β synthesis in the subchondral region, resulting in a progression of TMJ-OA
T. Kamiya et al. 2009 [26]	In vitro	TMJ porcine condyle cartilage cell isolated	Tensile loading: CG (unloaded) EG1 (7%) (12,24 and 48 h) EG1 (21%) (12,24,48 h)	Gene expression: Superficial zone protein (SZP), IL-1β, TGF-β1	EG1: SZP, IL-1β, TGF-β1 were upregulated after 12, 24 and 48 h EG2: SZP, IL-1β, TGF-β1 were upregulated on 12 h and decreased on 24 and 48 h	SZP is enhanced but optimal mechanical stimuli but inhibited by excessive loading, leading to an cartilage joint degradation by decreasing joint lubrication
S. Agarwal et al. 2001 [27]	In vitro	Isolated cartilage cells from rabbit TMJ discs	Tensile loading: CG = No loading + no IL-1β treatment EG1 = Loading 6% strain + no IL-1β treat. EG2 = No loading + IL-1β treat. EG3 = Loading 6% strain + IL-1β treat. For 48 and 96 h	Cellular response: Proteoglycan synthesis under different loading regimes and IL-1β treatment	Proteoglycans Synthesis: 48 h: EG2˂ (CG, EG1, EG3) 96 h:EG2˂(CG,EG1,EG3)	Application of cyclic tensile strain abrogated catabolic effects of IL-1β on TMJ chondrocytes.
S.-C. Su et al. 2014 [28]	In vitro	Isolated cells from porcine TMJ condyle cartilage	Tensile loading: CG (unloaded) EG1 (10%) (1,3,6,12,18,24 h) EG2 loading for 24 h + celecoxib	Cellular response: Cox-2, MMP-3,1 and 9; ADAMTS-5; PGE2 gene expression	Cox-2, MMP-3,1 and 9; ADAMTS-5 and PGE2 gene expression: CG˂EG1 EG1 6˂˂12˂18˂24 h EG1 ˃˃ EG2	Celecoxib exerts protective effects by decreasing degradation and restoring synthesis of extracellular matrix components.
H. Tabeian et al. 2016 [29]	In vitro	Isolated cells from porcine TMJ condyle cartilage	Tensile loading: CG (unloaded) EG1 (unloaded +TNF-α) EG2 (loaded – TNF-α treatment) EG3 (loaded + TNF-α treatment)	Cellular response: MMP-13, MMP-2, Coll IA, IIA, ACAN	MMP-13 gene expression: EG1˃ ˃ ˃ EG3	Cyclic tensile strain can protect the cartilage from inflammation.

*CG* control group, *EG* experimental group, *IHC* immuno-histochemical, *Micro CT* micro-computed tomography)

Twenty-six experimental studies were included in this systematic review: 15 were in vivo studies, of which 5 were dealing with changes in the hardness of diet and 10 were focusing on forced movement, and 11 were in vitro studies, of which 4 were dealing with compressive loading on the chondrocytes, 6 with tensile loading, and 1 with shear loading.

### Main findings

A wide variation of studies was included in this review. To enable sensible comparison of the results, several groups of studies were distinguished.

In the in vivo studies, different food consistencies, forced jaw movements (by the application of intraoral devices to restrict the jaw position or motion), or surgical intervention (e.g., osteotomies) were used to cause an alteration of the habitual mechanical loading, resulting in a change of the amplitude and/or direction of the TMJ loading. The effect of the different modes of mechanical loading was analyzed using several parameters. The response of the cartilage was assessed by analyzing either the anatomical structure (DJD like lesions) or the changes of the cellular response using microscopy and/or assessment of gene expression by polymerase chain reaction PCR.

In the in vitro studies, two types of studies can be identified. In the first type, chondrocytes were isolated from the cartilage and seeded on plates. These cells were then exposed to mechanical forces (compression, tensile, or shear). The response of these cells was determined by analyzing levels of gene expression by PCR. In the second type of studies, fresh pig TMJ discs were exposed to mechanical loading by means of compression, and the outcome measures were quantified as histological changes and alterations of the biomechanics properties of the disc.

## Discussion

### Strengths and limitations

This article aimed to identify the way in which different frequencies and magnitudes of mechanical loading can affect the fibrocartilage of the TMJ. Clear inclusion and exclusion criteria were used to select articles that would be suited to answer the aim. However, as the selected articles used different experimental designs, it was not possible to compare all the articles with each other. For this reason, the set of articles was split by type of study (in vivo and in vitro) and by how the loading was applied.

### Interpretation of the evidence

#### In vivo experiments

Excessive, repetitive loading can cause soft- and hard-tissue adaptation or degradation. This was shown when continuous static loading, such as forced mouth opening, was applied in vivo. After 1 day of mouth opening, a catabolic effect was noted: cartilage thickness decreased. The cartilage then adapted to this loading and reacted by increasing the synthesis of collagen type II and other elements of the extracellular matrix [16]. After 1 week of forced moth opening, DJD lesions were nevertheless found [12, 13, 19].

When the same forced mouth opening protocol was applied with different intensities, light forces provided remodeling of the TMJ, while heavy forces induced degeneration and maintained an inflammatory condition [15, 20].

In case of abnormal dynamic and static occlusal relationships, such as unilateral chewing and forced anterior cross-bite, the outcomes were always catabolic, with a decrease in the level of proteoglycans and collagen type II, and an increase in osteoclastic activity in the condyle [11, 14, 17]. It thus seems that functional overloading skews the balance between ECM formation and degradation in the TMJ towards the latter.

When the mechanical loading consisted of differences in diet hardness, a hard diet, leading to sufficient joint loading, induced an increase in the amount of collagen type II and chondrocyte maturation, thus indicating growth. A soft diet, resulting in a reduced joint loading, reduced cartilage thickness as well as the number of IGF-1 receptor positive cells, indicating reduced growth activity. These results support the importance of mechanical loading (such as chewing) as an essential stimulus to increase mandibular growth [4, 18]. TMJ loading through a hard diet was even able to increase collagen and aggrecan production and cartilage thickness when mechanical overloading was induced through forced mouth opening, thereby preventing cartilage degradation. The hypo function of the TMJ leads to DJD-like lesions [21].

Changes on the direction of the mechanical loading and condyle position after oblique vertical body osteotomy of the mandible and counterclockwise rotation, the same procedure used in Class II orthognathic surgery, induced idiopathic condylar resorption, a kind of DJD. This probably occurred because the trabecular bone patterns reflect the functional loading patterns during the growth period, and this change of condyle position and loading direction exposes an area that is less dense which could decrease the biomechanical properties needed to handle this loading [30].

Apart from loading, hormones may have an effect on the TMJ cartilage. Estrogen seems to inhibit the maturation of the chondrocytes and in cases in which a soft diet loading was applied and was expected to decrease cartilage thickness, such a catabolic effect was partially prevented by the lack of estrogen [25].

#### In vitro experiments

In vitro experiments showed that different types of loading regimes, such as tension, compression, and shear, had different effects on the TMJ cartilage chondrocytes when applied at low, moderate, or high intensity. At high intensity, tension and compression both caused a catabolic effect on the chondrocytes by reducing gene expression of the extracellular matrix components and increasing IL1-β production [26]. Unlike high intensity, low and moderate dynamic compression had an anabolic effect on the chondrocytes, increasing the expression of collagen type I and II and aggrecans [10]. These effects are time-dependent, as Nicodemus et al. [23] showed after application of dynamic compressive overloading. During the first 24 h, the gene expression of collagen type I and II and aggrecan increased, showing an adaptation behavior. After 48 h, the gene expression decreased to a level under the control levels, which demonstrates a catabolic effect of prolonged loading.

The reaction of TMJ disc-derived cells to compression is also time-dependent. When compression was applied for a short period and with longer intervals between cycles, fibrocartilage had more time to recover and return to the initial stage [9, 22]. This capacity to recover is changed when the collagen fiber network is disrupted, i.e., after a collagenase treatment as shown in fresh porcine discs [24]. Such a situation can occur in vivo in cases of intra-articular inflammation where cytokines stimulate degradation of collagen fibers. When shear movements were applied, the different parts of the TMJ disc reacted differently. The posterior zone was more resistant, with better biomechanical properties, and showed less deformation during loading than the anterior and intermediate zones of the disc [27].

In addition to a catabolic effect, cyclic tensile strain can also protect the cartilage from the effects of inflammation, e.g., suppressing the catabolic effect of TNF-α by down-regulating the expression of MMPs by TNF-α-treated chondrocytes [28, 29, 31]. As well as cyclic tensile strain, celecoxib has a protective effect by decreasing degradation and restoring synthesis of ECM in inflamed cartilage [32].

### Recommendations

More in vivo and in vitro studies in each type of study design are required to clarify how fibrocartilage reacts to different types of mechanical loading. In this regard, we would like to stress the importance of physical measurements of actual loading conditions in the tissues, as these can be quite different from what is assumed. For example, Rafferty et al. demonstrated that during mandibular distraction in minipigs, the increased cartilage thickness on the distraction side was associated with reduced rather than increased loading [33].

In addition, studies are needed to assess how mechanical loading could be incorporated in new protocols for the treatment of DJD, for example by including physiotherapy (e.g., cyclic loading). In vivo studies on the efficacy of orthognathic surgery on the TMJ would be important to predict side effects and to prevent idiopathic condyle resorption in patients.

The mechanical loading described in the included in vivo studies only includes diet and overloading by forced mouth opening and other artificial interventions, but it would be interesting to include other kinds of loading as well, mimicking clenching and grinding, and to assess how the TMJ reacts to these different intensities and frequencies of mechanical loading.

## Conclusion

Based on the studies included in this review, we could conclude that dynamic mechanical loading is an important stimulus for mandibular growth and for the homeostasis of TMJ cartilage. When this loading is applied at a low intensity, it protects inflamed cartilage by effectively antagonizing IL-1β. However, frequent overloading induces accelerated cell death and increased cartilage degradation.

## Electronic supplementary material

ESM 1 (DOCX 13 kb)

## References

[1] Yu D, Tiilikainen P, Raustia A, Pirttiniemi P (2007). Dietary loading and aggrecanase-1/TIMP-3 expression in rat mandibular condylar cartilage. J Orofac Pain.

[2] Chen W, Tang Y, Zheng M, Jiang J, Zhu G, Liang X, Li M (2013). Regulation of plasminogen activator activity and expression by cyclic mechanical stress in rat mandibular condylar chondrocytes. Mol Med Rep.

[3] Schlaak JF, Pfers I, Meyer Zum Büschenfelde KH, Märker-Hermann E (1996). Different cytokine profiles in the synovial fluid of patients with osteoarthritis, reumatoid arthritis and seronegative spondylarthropathies. Clin Exp Rheumatol.

[4] Hichijo N, Kawai N, Mori H, Sano R, Ohnuki Y, Okumura S, Langenbach GEJ, Tanaka E (2014). Effects of the masticatory demand on the rat mandibular development. J Oral Rehabil.

[5] O’Hara BP, Urban JP, Maroudas A (1990). Influence of cyclic loading on the nutrition of articular cartilage. Ann Rheum Dis.

[6] Copray JVM, Dibbets JMH, Kantoma T (1988). The role of condylar cartilage in the development of the temporomandibular joint. Angle Orthodon.

[7] Sun HB (2010). Mechanical loading, cartilage degradation, and arthritis. Ann N Y Acad Sci.

[8] Schiffman E, Ohrbach R, Truelove E, Look J, Anderson G, Goulet JP, List T, Svensson P, Gonzalez Y, Lobbezoo F, Michelotti A, Brooks SL, Ceusters W, Drangsholt M, Ettlin D, Gaul C, Goldberg L, Haythornthwaite J, Hollender L, Jensen R, John MT, De Laat A, De Leeuw R, Maixner W, van der Meulen M, Murray GM, Nixdorf DR, Palla S, Petersson A, Pionchon P, Smith B, Visscher CM, Zakrzewska J, Dworkin SF (2014). Diagnostic criteria for temporomandibular disorders (DC/TMD) for clinical and research applications: recommendations of the international RDC/TMD consortium network and orofacial pain special interest group. J Oral Facial Pain Headache.

[9] Carvalho RS, Yen EHK, Suga DM (1995). Glycosaminoglycan synthesis in the rat articular disk in response to mechanical stress. Am J Orthod Dentofac Orthop.

[10] Kamiya T, Tanimoto T, Tanne Y, Lin YY, Kunimatsu R, Yoshioka M, Tanaka N, Tanaka E, Tanne K (2010). Effects of mechanical stimuli on the synthesis of superficial zone protein in chondrocytes. J Biomed Mater Res A.

[11] Zhang M, Wang H, Zhang J, Zhang H, Yang H, Wan X, Jing L, Lu L, Liu X, Yu S, Chang X, Wang M (2016). Unilateral anterior crossbite induces aberrant mineral deposition in degenerative temporomandibular cartilage in rats. Osteoarthr Cartil.

[12] Soube T, Yeh W-C, Chhibber A, Ultreja A, Diaz-Doran V, Adams D, Wadhwa S (2011). Murine TMJ loading causes increased proliferation and chondrocyte maturation. J Dent Res.

[13] Fujisawa T, Kuboki T, Kasai T, Sonoyama W, Kojima S, Uehara J, Komori C, Yatani H, Hattori T, Takigawa M (2003). A repetitive, steady mouth opening induced an osteoarthritis-like lesion in the rabbit temporomandibular joint. J Dent Res.

[14] Poikela A, Kantomaa T, Perttiniemi P, Tuukkanen J, Pietilä K (2000). Unilateral masticatory funcition chnages the proteoglycan content of mandibular condylar cartilage in rabbit. Cells Tissues Organs.

[15] Kartha S, Zhou T, Granquist EJ, Winkelstein BA (2016) Development of a rat model of mechanically induced tunable pain and associated temporomandibular joint responses. J Oral Maxillofac Surg 74:54.e1–54.e10 10.1016/j.joms.2015.09.005 26433038

[16] Utreja A, Yadav S, Villa MM, Li Y, Jiang X, Nanda R, Rowe DW (2016). Cell and matrix response of temporomandibular cartilage to mechanical loading. Osteoarthr Cartil.

[17] Liu Y-D, Liao L-F, Zhang H-Y, Lu L, Jiao K, Zhang M, Zhang J, He J-J, Wu Y-P, Chen D, Wang M-Q (2014). Reducing dietary loading decreases mouse temporomandibular joint degradation induced by anterior crossbite prosthesis. Osteoarthr Cartil.

[18] Ravosa MJ, Kunwar R, Stock SR, Stack MS (2007). Pushing to the limit: masticatory stress and adaptative plasticity in mammalian craniomandibular joints. J Exp Biol.

[19] Fujimura K, Kobayashi S, Susuki T, Segami N (2005). Histologic evaluation of temporomandibular arthritis induced by mild mechanical loading in rabbits. J Oral Pathol Med.

[20] Zhang C, Xu Y, Cheng Y, Wu T, Li H (2015). Effect of asymmetric force on the condylar cartilage, subchondral bone and collagens in the temporomandibular joints. Arch Oral Biol.

[21] Ikeda Y, Yonemitsu I, Takei M, Shibata S, Ono T (2014). Mechanical loading leads to osteoarthritis-like changes in the hypofunctional temporomandibular joint in rats. Arch Oral Biol.

[22] Nickel JC, Iwasaki LR, Beatty MW, Marx DB (2004). Laboratory stresses and tractional forces on the TMJ disc surface. J Dent Res.

[23] Nicodemus GD, Villanueva I, Bryant SJ (2007). Mechanical stimulation of TMJ condylar chondrocytes in PEG hydrogels. J Biomed Mater Res A.

[24] Fazaeli S, Ghazanfari S, Everts V, Smit TH, Koolstra JH (2016). The contribution of collagen fibers to the mechanical compressive properties of the temporomandibular joint disc. Osteoarthr Cartil.

[25] Orajärvi M, Puijola E, Yu S-B, Liu X, Tiilikainen P, Wang M, Raustia A, Perttiniemi P (2012). Effect of estrogen and dietary loading on condylar cartilage. J Orofac Pain.

[26] Lin Y-Y, Tanaka N, Ohkuma S, Kamiya T, Kunimatsu R, Huang Y-C, Yoshioka M, Mitsuyoshi T, Tanne Y, Tanimoto K, Tanaka E, Tanne K (2009). The mandibular cartilage metabolism is altered by damaged subchondral bone from traumatic impact loading. Ann Biomed Eng.

[27] Juran CM, Dolwick MF, McFetrige PS (2013). Shear mechanics of the TMJ disc: relationship to common clinical observations. J Dent Res.

[28] Tabeian H, Bakker AD, Betti BF, Lobbezoo F, Everts V, de Vries TJ (2016). Cyclic tensile strain reduces TNF-α induced expression of MMP-13 by condylar temporomandibular joint cells. J Cell Physiol.

[29] Deschner J, Rath-Deschner B, Agarwal S (2006). Regulation of matrix metalloproteinase expression by dynamic tensile strain in rat fibro chondrocytes. Osteoarthr Cartil.

[30] Yang HJ, Hwang SJ (2014). Osseous alterations in the condylar head after unilateral surgical directional change in rabbit mandibular condyles: preliminary study. J Craniomaxilofacial Surg.

[31] Agarwal S, Long P, Gassner R, Piesco NP, Buckley MJ (2001). Cyclic tensile strain suppresses catabolic effects of interleukin-1β in fibrochondrocytes from the temporomandibular joint. Arthritis Reum.

[32] Su S-C, Tanimoto K, Tanne Y, Kunimatsu R, Hirose N, Mitsuyoshi T, Okamoto Y (2014). Celocoxib exerts protective effects on extracellular matrix metabolism of mandibular condylar chondrocytes under excessive mechanical stress. Osteoarthr Cartil.

[33] Rafferty KL, Sun Z, Egbert M, Bakko DW, Herring SW (2007). Changes in growth and morphology of the condyle following mandibular distraction in minipigs: overloading or underloading. Arch Oral Biol.

