# Supplementary material for: Effect of mechanical loading on the metabolic activity of cells in the temporomandibular joint: a systematic review
Source: Clin Oral Investig. 2017 Aug 1;22(1):57–67. doi: 10.1007/s00784-017-2189-9 (PMC5748425; doi:10.1007/s00784-017-2189-9)
Supplement: Supplementary file 1 — (DOCX 13 kb) [file 784_2017_2189_MOESM1_ESM.docx]

**SUPPLEMENTARY INFORMATIONSEARCH STRATEGY**

**Search strategy for Embase.com (September 20, 2016)**

/exp = EMtree keyword with explosion

/de = EMtree keyword without explosion

:ab,ti = words in title or abstract

NEAR/x = words near to each other, x places apart

| **Search** | **Query** | **Items found** |
| --- | --- | --- |
| **#6** | #1 AND #2 AND #5 | **182** |
| **#5** | 'temporomandibular joint'/exp OR tmj:ab,ti OR tmjs:ab,ti OR (temporomandib*:ab,ti OR craniomandib*:ab,ti OR mandib*:ab,ti OR jaw:ab,ti OR jaws:ab,ti OR mandibulotempor*:ab,ti AND (joint*:ab,ti OR disk*:ab,ti OR disc*:ab,ti OR articular*:ab,ti OR menisc*:ab,ti)) | **30,604** |
| **#2** | 'cartilage'/exp OR cartilage*:ab,ti OR fibrocartilage*:ab,ti | **112,859** |
| **#1** | 'shear strength'/exp OR 'tensile strength'/exp OR 'compressive strength'/exp OR 'mastication'/exp OR 'mechanical stress'/de OR 'shear stress'/exp OR 'hydrostatic pressure'/exp OR (masticatory NEAR/3 force*):ab,ti OR (bite NEAR/3 force*):ab,ti OR (occlusal NEAR/3 force*):ab,ti OR (tensil*:ab,ti OR shear*:ab,ti OR compressi*:ab,ti AND strength*:ab,ti) OR (mechanical NEAR/3 stress*):ab,ti OR hydrostatic*:ab,ti OR loading:ab,ti OR load:ab,ti OR loads:ab,ti | **352,121** |

**Search strategy for Thomson Reuters/Web of Science (September 20, 2016)**

TOPIC = words in title, abstract or keywords

| **Search** | **Query** | **Items found** |
| --- | --- | --- |
| **#1** | **TOPIC:** (((masticatory AND force*) OR (bite AND force*) OR (occlusal AND force*) OR ((tensil* OR shear* OR compressi*) AND strength*) OR (mechanical AND stress*) OR hydrostatic* OR loading OR load OR loads) AND (cartilage* OR fibrocartilage*) AND (tmj OR tmjs OR ((temporomandib* OR craniomandib* OR mandib* OR jaw OR jaws OR mandibulotempor*) AND (joint* OR disk* OR disc* OR articular* OR menisc*)))) | **225** |

**Search strategy for PubMed (September 20, 2016)**

[Mesh] = Medical subject headings (MeSH)

[tiab] = words in title or abstract

| **Search** | **Query** | **Items found** |
| --- | --- | --- |
| **#4** | #1 AND #2 AND #3 | **254** |
| **#3** | "Tensile Strength"[Mesh] OR "Compressive Strength"[Mesh] OR "Shear Strength"[Mesh] OR "Bite Force"[Mesh] OR "Stress, Mechanical"[Mesh] OR "Hydrostatic Pressure"[Mesh] OR masticatory force*[tiab] OR bite force*[tiab] OR occlusal force*[tiab] OR ((tensil*[tiab] OR shear*[tiab] OR compressi*[tiab]) AND strength*[tiab]) OR mechanical stress*[tiab] OR hydrostatic*[tiab] OR loading[tiab] OR load[tiab] OR loads[tiab] | **334,432** |
| **#2** | "Cartilage"[Mesh] OR cartilage*[tiab] OR fibrocartilage*[tiab] | **108,288** |
| **#1** | "Temporomandibular Joint"[Mesh] OR tmj[tiab] OR tmjs[tiab] OR ((temporomandib*[tiab] OR craniomandib*[tiab] OR mandib*[tiab] OR jaw[tiab] OR jaws[tiab] OR mandibulotempor*[tiab]) AND (joint*[tiab] OR disk*[tiab] OR disc[tiab] OR discs[tiab] OR articular*[tiab] OR menisc*[tiab])) | **21,111** |
